# The Role of the HIF-1α Transcription Factor in Increased Cell Division at Physiological Oxygen Tensions

**DOI:** 10.1371/journal.pone.0097938

**Published:** 2014-05-16

**Authors:** Samantha Carrera, Joana Senra, Maria Isabel Acosta, Mohammad Althubiti, Ester M. Hammond, Petra J. de Verdier, Salvador Macip

**Affiliations:** 1 Department of Biochemistry, University of Leicester, Leicester, United Kingdom; 2 Cancer Research U.K./MRC Gray Institute for Radiation Oncology and Biology, Department of Oncology, University of Oxford, Oxford, United Kingdom; 3 Karolinska Institutet, Department of Molecular Medicine and Surgery, Urology Laboratory, and Department of Urology, Karolinska University Hospital, Stockholm, Sweden; University of Dundee, United Kingdom

## Abstract

HIF-1 is a transcription factor that mediates the cellular responses to low oxygen environments, mainly as a result of having an oxygen-labile subunit, HIF-1α. HIF-1α has been carefully studied in the context of severe hypoxic stresses (<1% O_2_), but it is also known to be present at oxygen tensions commonly found in normal tissues *in vivo* (∼1–13% O_2_), albeit at much lower levels. Its role under these physiological conditions is not fully understood. Here, we show that a transcriptionally active HIF-1α was up-regulated at 5% O_2_, both in normal and cancer cells, but only some of its target genes were elevated as a result. HIF-1α induction was in part dependent on the activation of the ERK1/2 MAPK signalling pathway, which we have previously shown is active at 5% O_2_. We also found that HIF-1α does not contribute to the protection against DNA damage that can be observed in low oxygen environments, and that there are certain DNA damaging agents, such as doxorubicin and actinomycin D, that prevent HIF-1α induction independently of p53. Moreover, absence of HIF-1α significantly reduced the growth advantage of cells cultured at 5% O_2_. In view of these data, we conclude that HIF-1α can be induced and activated at physiological oxygen tensions in a MAPK-dependent manner and that, although this does not lead to pro-survival responses to stress, it determines the increased cell proliferation rates that are common under these conditions.

## Introduction

Although culturing cells under atmospheric oxygen tensions (∼20% O_2_) is a common laboratory practice, recent studies suggest that this seriously compromises their replicative lifespan. For instance, it has been shown that both human and mouse fibroblasts grown at levels of oxygen that mimic those commonly found *in vivo* (3% O_2_) undergo a higher number of cell divisions before becoming senescent [1], [2]. Consistent with this, human mesenchymal stem cells exhibited a decreased rate of telomere shortening and oxidative phosphorylation in these conditions [3]. For reasons that are not completely understood, these increases in replicative lifespan are typically associated with elevated rates of cell proliferation. Moreover, we have shown that cells cultured at physiological oxygen tensions up-regulate factors that protect them against DNA damaging agents [4]. All these data together suggest that, in response to the concentrations of oxygen normally available in tissues, cells may be constitutively subjected to a series of signals that favour their growth and survival. Due to the nature of prevalent culture techniques, these pathways have not been carefully studied.

HIF-1 is a transcription factor induced in response to low oxygen levels [5]. It binds to the RCGTG pentanucleotide present in the specific sequences of target genes, known as the hypoxia responsive element (HRE), and activates over a hundred genes that promote adaptation and survival [6]. HIF-1 is a heterodimeric complex composed of two subunits: the constitutively expressed HIF-1β and HIF-1α, which is sensitive to oxygen. When high concentrations of oxygen are present, prolyl hydroxylase domain (PHD) enzymes hydroxylate HIF-1α, allowing it to be recognized by pVHL and targeted for proteasomal degradation [7]. A growing body of evidence suggests that HIF-1 can contribute to tumour progression and metastasis [8]. Indeed, many of the signalling pathways that are deregulated in cancer, such as MAPK or Wnt/β-catenin, promote its activity [9]–[11]. Moreover, pVHL mutations in renal cell carcinoma block the degradation of HIF-1α [12]. As a result of all this, HIF-1α is overexpressed in a variety of tumours, usually being a marker of poor prognosis, early recurrence and resistance to chemotherapy [13].

In normal tissues, HIF-1α has been extensively studied in the context of cellular stress responses to the severe reduction in oxygen levels known as hypoxia (0.1–1% O_2_). However, because its expression is inversely proportional to the levels of oxygen present in the microenvironment, it can also be detected in a range of physiologically relevant non-stressful tensions [14], sometimes referred to as physioxia or physoxia (∼1–13%) [15]. The roles of HIF-1α in these environments are still debated. Here, we characterize the expression and function of HIF-1α at 5% O_2_, which we chose as a representative oxygen tension found in several human tissues [15]. We show that although HIF-1α is expressed at much lower levels than under hypoxic stresses, it is still transcriptionally active and contributes to an increase in cell proliferation.

## Materials and Methods

### Cell Culture

Colon cancer cell lines HCT116 (American Type Culture Collection, Manassas, VA), breast cancer cell line MCF-7 (American Type Culture Collection, Manassas, VA) and normal human keratinocytes cells (a gift from K. Herbert, University of Leicester, Leicester, UK, unpublished) were maintained in DMEM supplemented with 10% FBS and penicillin-streptomycin (50 units/ml). Fresh medium was added at least every three days. Doxorubicin (Sigma-Aldrich), U0126 (Promega), AZ6244 (Selleck Chemicals), Actinomycin D, tert-Butyl Hydroperoxide (Sigma-Aldrich), YC-1 (Cayman Chemical) and CoCl_2_ (Sigma-Aldrich) were added to the culture media and were not removed until analysis was performed, or when media was changed, as specified. Cells were counted with a Bio-Rad TC-20 automated cell counter. Incubations at 5% O_2_ were performed in a Sanyo MCO-5M cell incubator. Hypoxia was achieved by culturing cells for the specified times inside a Hypoxia Incubator Chamber (Stemcell technologies) flushed with N_2_ through a flow meter. Passage of cells was always carried at atmospheric oxygen tensions.

### Inhibition of HIF-1α Expression

Cells were transfected with a specific HIF-1α siRNA (Santa Cruz, sc-35561) or a luciferase siRNA (control). Transfections were performed with Lipofectamine 2000 (Invitrogen), following manufacturer’s specifications, two days before treating the cells with other drugs.

### FACS Analysis

Cells were washed with PBS and collected after treatment. Cell pellets were fixed using 1 ml of 70% ethanol and placed at −20°C for at least 30 min. Cells were then stained with 500 µl of PI buffer (50 µg/ml of Propidium Iodide, 10 µg/ml RNase A, 1×PBS) and transferred to 3.5 ml polystyrene round-bottom tubes (VWR). The tubes were incubated for 30 min at 37°C in the dark. 10,000 events were recorded for each sample using the Beckton Dickinson FACSCanto II and FACSDiva 6.0 software (Beckton Dickinson) for acquisition and analysis.

### Immunoblotting

Cells were washed once with ice-cold 1x PBS and lysed using 500 µl of ProteoJET Mammalian Cell Lysis Reagent (Fermentas). Cells were scraped and collected in a 1.5 ml microcentrifuge tube. 5 µl of Protease Inhibitor Cocktail Set III (Calbiochem) and 1 µM Na_3_VO_4_ (Sigma-Aldrich) were added to each sample. Lysates were cleared by centrifugation at maximum speed for 15 min. Total protein concentration was quantified using Bradford reagent (Fermentas). 20 µg of total cell protein were subjected to 10% SDS-PAGE and transferred to an Immobilon-P membrane (Millipore). Antibodies against HIF-1α (BD Transduction Laboratories, #610958), HIF-2α (Novus biologicals, #NB100–132), Glut-1 (Abcam, #ab652), PHD2 (Abcam, # ab4561), ERK 1/2 (Cell Signalling, #9102), P-ERK 1/2 (Cell Signalling, #9101), p53 (Abcam, #ab28) and p21 (Santa Cruz, #sc-397) were used. β-actin (Abcam, #ab3280, or Santa Cruz Biotechnology, #sc-69879) was used as a loading control. The detection of the proteins was made using Pierce ECL plus western blotting substrate (Thermo Scientific).

### Quantitative Real Time PCR (qRT-PCR)

Total RNA was extracted using TRIzol (Invitrogen), following manufacturer’s protocols. RNA was resuspended in RNase-free water by passing the solution a few times through a pipette tip. Total RNA was quantified using a Nanodrop ND8000 (Thermo Scientific). cDNA was prepared from 1 µg of RNA with the Precision qScript Reverse Transcription kit (PrimerDesign) using oligo-dT primers, following manufacturer’s instructions. The cDNA was diluted 1∶10 and 5 µl were used for each reaction. Experiments were all performed in triplicate. Custom designed real-time PCR assay from PrimerDesign Ltd was used for the qRT-PCR with 2×Precision Mastermix (PrimerDesign Ltd), following manufacturer’s instructions. Reactions were carried out on a Roche Light Cycler 480 under the following conditions: enzyme activation for 10 minutes at 95°C, followed by 50 cycles of denaturation for 15 seconds at 95°C and data collection for 60 seconds at 60°C. A post PCR run melting curve was used to prove the specificity of the primers. Primers: GLUT1 (ACCTCACTCCTGTTACTTACCTA, ACCCCACTTACTTCTGTCTCA), HIF-1α (TGCCACATCATCACCATATAGAG, TGACTCAAAGCGACAGATAACA), PGK1 (TGCCCATGCCTGACAAGTA, CTACACAGTCCTTCAAGAACAGA), VEFG (CCAGGAAAGACTGATACAGAACG, GGTTTCTGGATTAAGGACTGTTC).

### Luciferase Assay

Cells were grown into 24-well plates and transfected with a pGL3 Promoter vector containing trimers of the HREs of the Phosphoglycerate kinase 1 (PGK-1) gene (generously provided by Dr Burke, University of Leicester) [16] and co-transfected with a β-galactosidase plasmid (transfection control). Medium was removed from the wells and cells were washed once with PBS. 140 µl of ProteoJET Mammalian Cell Lysis Solution (Fermentas) was added to each well and plates were incubated on a shaker for 2 hours at room temperature. For β-galactosidase, 80 µl of each cell lysate were transferred into a 96-well plate and 100 µl of β-galactosidase substrate was added to each well and incubated at 37°C for 15 minutes. The absorbance was measured at 405 nm. For the luciferase assay, 20 µl of each cell lysate were transferred into a white 96-well plate. 50 µl of luciferase substrate (Promega) was dispensed into each well and light emission measurements were taken after 30 seconds. Luciferase activity was normalized to β-galactosidase activity.

### Immunofluorescence

Cells were split into 6-well plates containing sterile coverslips. Cells were incubated with EdU (Molecular Probes, Life technologies) for 30 minutes and stained with the Click-iT EdU Imaging kit (Molecular Probes, Life technologies) following manufacturer’s instructions. Coverslips were washed three times with 1×PBS and stained with 4',6-Diamidino-2-Phenylindole, Dihydrochloride (DAPI, Invitrogen) for 10 minutes. Slides were labelled and the coverslips were mounted and sealed with transparent nail varnish. Slides were analysed using a Nokia TE300 semi-automatic microscope.

## Results

### Activation of HIF-1 Signalling at Physiological Oxygen Tensions

**Figure 1:**
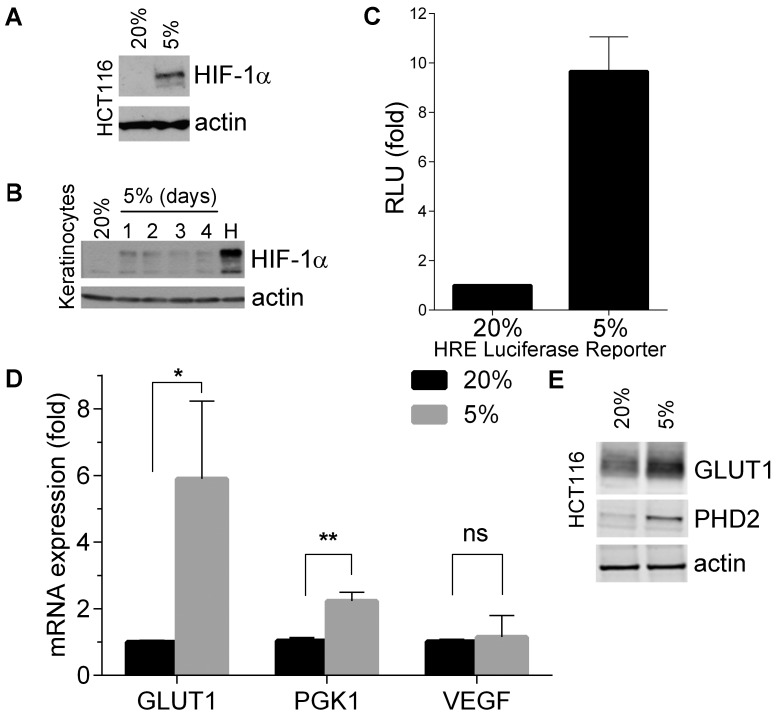
Physiological oxygen tensions induce HIF-1α expression and activity. (**A**) Western blot showing the protein levels of HIF-1α in HCT116. Cells were cultured at 20% or 5% O_2_ for 72 hours. (**B**) Western blot showing the protein levels of HIF-1α in normal human keratinocytes cultured for 1 to 4 days at 5% O_2_ or treated 500 µM of chemical hypoximimetic CoCl_2_ for 16 hours (H). (**C**) Luciferase assay showing HIF-1α in HCT116 cells. HCT116 were transfected with a PGK-1 luciferase reporter plasmid and a β-galactosidase control plasmid and then cultured for 48 hours at 5% O_2_. β-galactosidase activity was used to normalize luciferase activity. Luciferase activity is expressed as a ratio to 20% O_2_ levels. Results show mean values of 3 independent experiments and error bars represent standard deviation (**D**) qRT-PCR showing mRNA levels of Glut-1, PGK-1 and VEGF in HCT116 cells cultured at 20% and 5% O_2_ for 24 hours. Results show mean values of 3 independent experiments and error bars represent standard deviation. P values (unpaired t-tests): 0.03 (*), 0.001 (**), 0.7 (ns). (E) Western blot of lysates of HCT116 cultured at 20% or 5% O_2_ for 72 hours, showing expression of Glut-1 and PHD2.

To confirm that HIF-1α is induced under physiological conditions [14], we first cultured the colon cancer cell line, HCT116, at 5% O_2_, an oxygen tension found *in vivo* in tissues such as lung, brain, skin, liver and venous blood [15], [17]. As shown in Figure 1A, HIF-1α protein levels increased in these cells when compared to those cultured at atmospheric oxygen concentrations (20%), while levels of HIF-2α did not change (Figure S1A). This was also observed in normal human keratinocytes and was maintained even in prolonged cultures at 5% O_2_ (Figure 1B). Consistently with previously published observations [14], the amount of HIF-1α induced was inversely proportional to the levels of oxygen present.

To test whether the moderate levels of HIF-1α protein observed at 5% O_2_ were sufficient to provide a HIF-1-related response, we used a reporter plasmid driven by the HRE of PGK-1, a well-known HIF-1 target gene [16]. Figure 1C shows that HIF-1α was indeed active at 5% O_2_. This was confirmed by measuring the mRNA levels of HIF-1 target genes Glut-1, PGK-1 and VEGF by qPCR (Figure 1D). Although VEGF levels did not increase, both PGK-1 and Glut-1 showed a significant up-regulation at 5% O_2_. As expected, protein levels of Glut-1 and PHD2 (another HIF-1 target) were also observed to increase in a Western blot (Figure 1E). We concluded that HIF-1α can be induced at physiological oxygen tensions in normal and cancer cells, and that this basal expression is sufficient to activate the transcription of at least some of its target genes.

### MAPK Contributes to the Expression of HIF-1α at Physiological Oxygen Tensions

**Figure 2:**
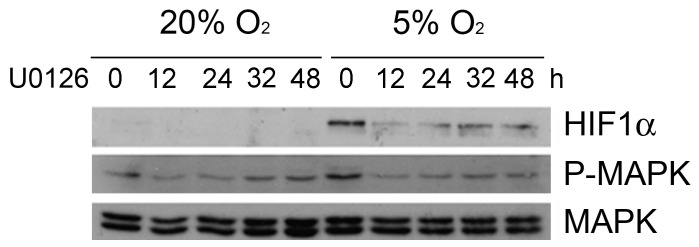
Chemical inhibition of MAPK reduces the activation of HIF-1α. Western blot showing the protein levels of HIF-1α and phosphorylated (active) ERK 1/2 MAPK in HCT116 cultured at 20% or 5% O_2_for 12 to 48 hours, in the presence of 1.25 µM U0126. U0126 was added at the same time cells were transferred to 5%O_2_. Total MAPK levels are provided as loading control.

It has been suggested that the ERK1/2 MAPK can induce, phosphorylate and stabilize HIF-1α, and thus promote its transcriptional activity [9], [11]. Moreover, MAPK signalling may have an effect on the interaction of p300 with HIF-1α, which is required for transactivation of target genes [18]. Also, we have previously shown that ERK1/2 MAPK is constitutively active at 5% O_2_ [4]_._ In view of all this information, we tested whether MAPK contributed to the up-regulation of HIF-1α at physiological oxygen tensions. As shown in Figure 2, blocking MAPK signalling with a chemical MEK inhibitor resulted in a significant reduction of the levels of HIF-1α protein induced at 5% O_2_. HIF-1α suppression was already evident 12 hours after treatment and was maintained even after prolonged exposure to U0126. Although the main top HIF-1α band was eliminated, faint smaller bands could still be observed in the blot. Moreover, expression of HIF-1α target gene Glut-1 at 5% O_2_ was also reduced 12 and 24 hours after inhibition of MAPK signalling (Figure S1B), confirming that HIF-1α transcriptional activity was being affected by the MEK inhibitors. These data suggest that the up-regulation of HIF-1α at physiological oxygen tensions is highly dependent on MAPK signalling in the HCT116 cancer cell line.

### Role of HIF-1α in the Response against DNA Damage at Physiological Oxygen Tensions

**Figure 3:**
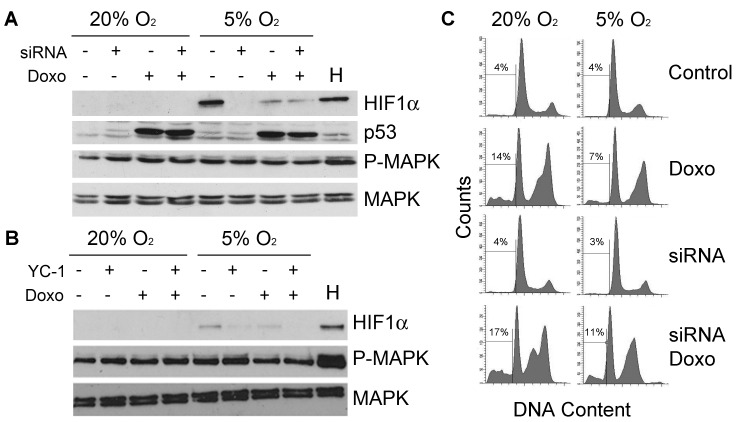
HIF-1α has no effect on the activation of MAPK at physiological oxygen tensions. (**A**) Western blot showing the protein levels of HIF-1α, p53, phosphorylated ERK 1/2 (P-MAPK) and ERK 1/2 (MAPK) in HCT116 cells transfected with 200 pmol of siRNA against HIF-1α and treated with doxorubicin (0.4 µg/ml). Control cells were transfected with a luciferase siRNA instead. Cells treated with 500 µM CoCl_2_ for 16 hours were used as a positive control for the induction of HIF-1α (H). (**B**) Western blot showing the protein levels of HIF-1α, phosphorylated ERK 1/2 (P-MAPK) and ERK 1/2 (MAPK) in HCT116 treated with 40 µM YC-1 and/or 0.4 µg/ml doxorubicin and cultured at 20% or 5% O_2_ for 24 hours. Cells treated with 500 µM CoCl_2_ for 16 hours were used as a positive control for the induction of HIF-1α (H). (**C**) Representative FACS plots of HCT116 cells stained with PI. Cells were transfected with 50 pmol of siRNA against HIF-1α and treated with 0.4 µg/ml doxorubicin. Cells were cultured at 20% or 5% O_2_ for 24 hours. Percentages indicate number of subG_1_ events (dead cells).

We have previously shown that the presence of MAPK signalling at 5% O_2_ reduces the apoptotic response to genotoxic stress [4]. Since HIF-1 has been reported to induce MAPK during hypoxia [19], we hypothesized that it could also be contributing to its activation at physiological oxygen tensions and thus helping protect cells against damage. This would imply a positive feedback loop between HIF-1α and MAPK that would enhance pro-survival signals. However, blocking HIF-1α expression with either a specific siRNA (Figure 3A) or the chemical inhibitor, YC-1 (Figure 3B), did not affect the levels of phosphorylated MAPK in the presence or absence of DNA damage. Of note, the absence of HIF-1α did not interfere with the induction of p53, one of the main triggers of apoptosis [20], in response to DNA damaging agent doxorubicin. Consistent with this data, HIF-1α inhibition did not reverse the protection against doxorubicin (Figure 3C). We concluded that the HIF-1α expression observed at physiological oxygen tensions does not have a significant effect in activating MAPK or protecting cells against genotoxic stresses.

### Inhibition of HIF1α by DNA Damaging Agents

**Figure 4:**
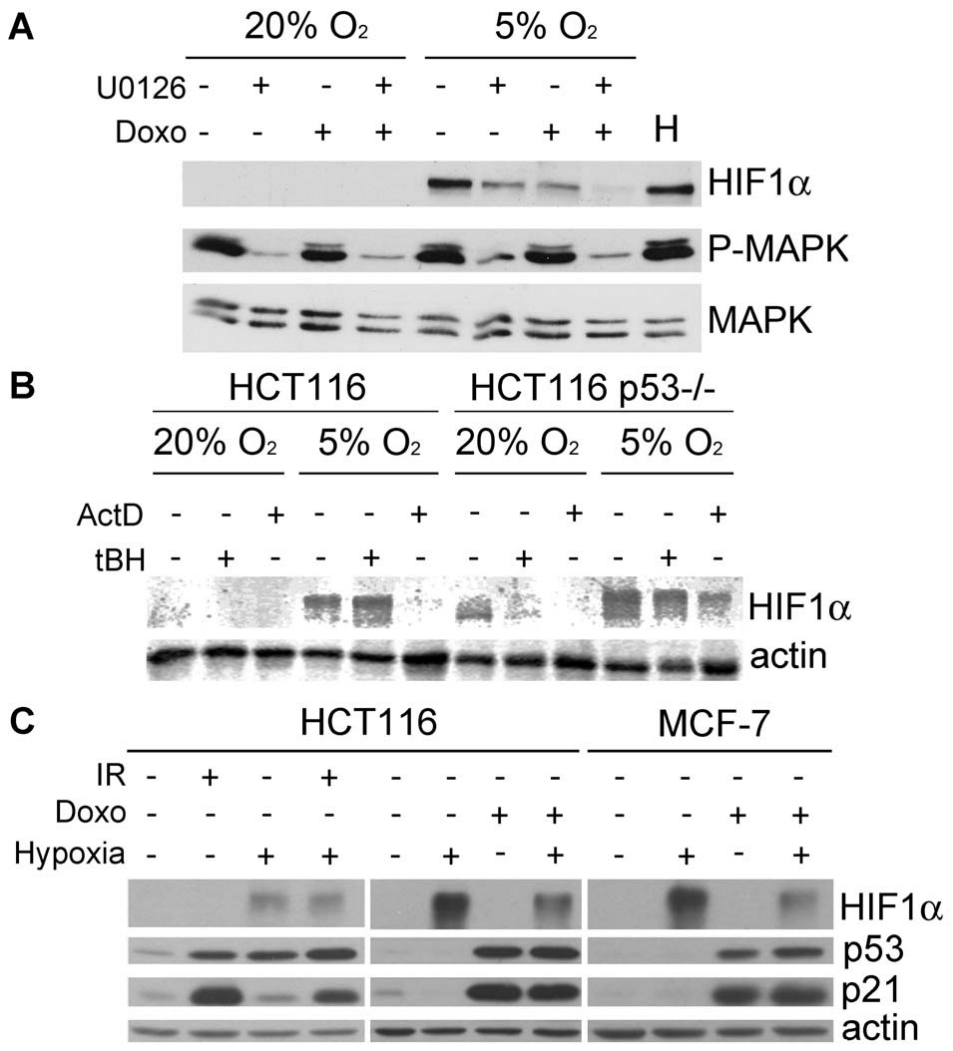
Inhibition of HIF-1α expression by DNA damaging agents. (**A**) Western blot showing the protein levels of HIF-1α, phosphorylated ERK 1/2 (P-MAPK) and ERK 1/2 (MAPK) in HCT116 cells treated with 1.25 µM U0126 and 0.4 µg/ml doxorubicin, and cultured at 20% or 5% O_2_ for 2 days. Cells treated with 500 µM CoCl_2_ for 16 hours were used as a positive control for the induction of HIF-1α (H). (**B**) Western blot showing the protein levels of HIF-1α in HCT116 and HCT116 p53^−/−^ in lysates collected 24 hours after treatment with 1 µg/ml Actinomicyn D (ActD) or 200 µM tert-Butyl Hydroperoxyde (tBH) for 2hours (**C**) Western blot showing the protein levels of HIF-1α, p53 and p21 in HCT116 and MCF-7 cells treated with 0.4 µg/ml doxorubicin or 10 Gy γ-radiation for 24 hours at 20% or <1% O_2_ (hypoxia).

Figures 3A and B unexpectedly showed that treatment with doxorubicin supressed the induction of HIF-1α at 5% O_2_. Further experiments showed that doxorubicin had an effect on HIF-1α similar to that observed with a MEK inhibitor, and both chemicals had an additive effect, totally suppressing HIF-1α expression (Figure 4A). Although it has been proposed that doxorubicin and other anthracycline antibiotics can block the activity of HIF-1 by interfering with its binding to DNA [21], our data suggest that they may also affect the induction and/or stability of the protein. Indeed, we observed no increase in the levels of HIF-1α mRNA, suggesting that the effects of 5% O_2_ were mostly on protein stability (Figure S1C). We next tested whether this effect was also observed after treatment with other DNA damaging agents. As shown in Figure 4B, the genotoxic antibiotic Actinomycin D also inhibited HIF-1α in HCT116, but that was not the case with the oxidant tert-butyl hydroperoxide (tBH). It is known that p53 can inhibit HIF-1α expression in certain situations [22]–[24]. However, an isogenic HCT116 line that lacks p53 showed the same response, suggesting that it was a p53-independent effect.

Finally, we observed that DNA damage reduced HIF-1α not only at physiological oxygen tensions but also when it was induced by hypoxic stress. As shown in Figure 4C, doxorubicin was able to block HIF-1α expression in different cell lines exposed to O_2_ concentrations below 1%. However, the same effect was not observed after treatment with ionizing radiation. These data together suggest that certain DNA damaging agents, but not all, have the ability to block HIF-1α at different oxygen tensions and that this is independent of the damage-mediated activation of the p53 pathway.

### Contribution of HIF-1α to Cell Proliferation

**Figure 5:**
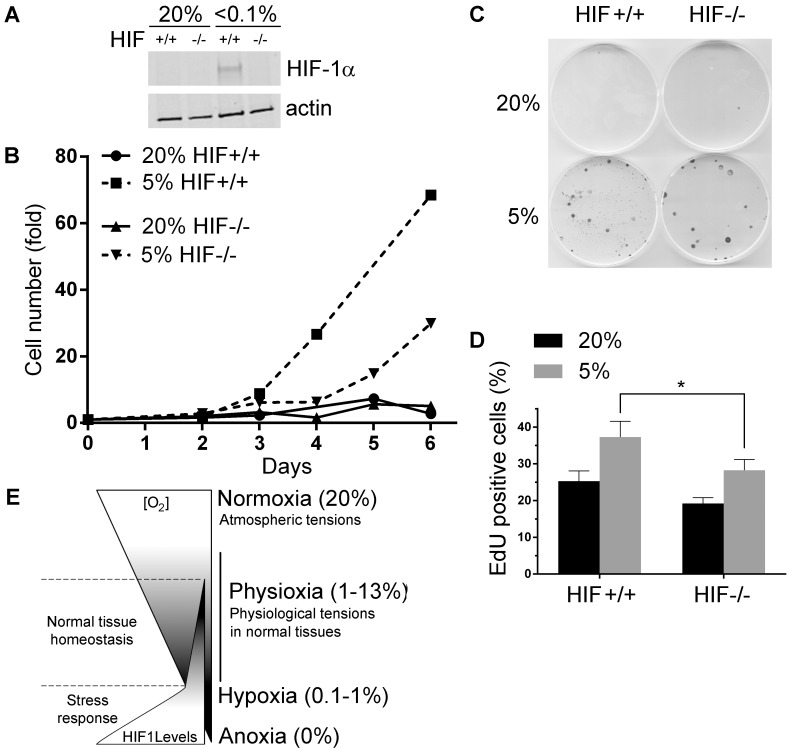
HIF-1α contributes to increased proliferation of cells at physiological oxygen tensions. (**A**) Western blot showing the protein levels of HIF-1α in HCT116 HIF^+/+^ and HIF^−/−^cultured either at 20% O_2_ or under hypoxic stress (<0.1% O_2_) for 16 hours. (**B**) Proliferation curves of HIF^+/+^ and HIF^−/−^ HCT116 cells cultured at 20% or 5% O_2_ from 2 to 8 days. Values represent ratio of cell numbers normalized to the initial seeded cells (10^6^). (**C**) Representative colony formation assay for HCT116 HIF^+/+^ and HIF^−/−^cultured at 20% or 5% O_2_. 200 cells were seeded in each plate and 14 days later they were stained with Giemsa. Media was not changed during the process. (**D**) Percentage of EdU positive HCT116 HIF^+/+^ and HIF^−/−^ cells as assessed by immunofluorescence (see Figure S2). Cells were incubated with EdU for 30 minutes in the corresponding oxygen tensions. Results represent means of two independent experiments. Two microscope fields were scored in each experiment. Error bars represent standard error. P value (unpaired t-test): 0.0127 (*), (**E**) Proposed model of the roles of HIF-1 at different oxygen concentrations.

To further study the role of HIF-1α physiological oxygen tensions, we asked whether it contributed to the increased cell proliferation usually observed under these conditions [3], [4]. To this end, we used an isogenic HCT116 cell line that lacks HIF-1α expression (Figure 5A) [25]. Figure 5B shows that control HCT116 cells proliferated better at 5% than 20% O_2_ (circles vs. squares), as expected. This was also the case in the absence of HIF-1α (up triangles vs. down triangles), but the difference then was only half of that observed when HIF-1α was present. This was confirmed with a colony formation assay that showed higher cell proliferation at 5% O_2_, which was only partially reduced in the absence of HIF-1α (Figure 5C). Moreover, EdU staining showed a higher number of cells undergoing division at 5% O_2_, although with a significant reduction in cells that do not express HIF-1α (Figure 5D and Figure S2). All these data together indicates that HIF-1α contributes proliferation signals at physiological oxygen levels that encourage an increased rate of cell division, although it is not the only determinant factor for this effect.

## Discussion

HIF-1α, one of the main regulators of the cellular responses to reduced oxygen tensions, is targeted for proteasomal degradation by PHDs when oxygen concentrations are above 100 µM [26]. Most normal tissues under physiological conditions (physioxia) only have ∼10–30 µM O_2_ [27], which suggests that HIF-1α should be constitutively expressed in a great proportion of cells in the human body (Figure 5E). Indeed, HIF-1α has been detected at a range of biologically relevant oxygen concentrations [14]. This is consistent with a possible role of HIF-1α in normal cellular homeostasis, which has not been explored in detail, in contrast with its well-known activity as a stress-response and tumour promoter factor.

Our results show that HIF-1α was present at low levels and functional at oxygen tensions commonly found *in vivo* (5% O_2_), although not all its targets genes explored were up-regulated. The fact that HIF-1α is active even when cells are not under a stressful oxygen deprivation supports the hypothesis of its involvement in the basal physiological processes of the cell. It is likely that, similar to what happens to other transcription factors [28], the amount of HIF-1α protein expressed determines the induction of a particular set of genes due to a different affinity to their promoters. This would define a series of distinct adaptive responses depending on the microenviroment. In fact, it has been previously proposed that HIF-1 may be rapidly activated at lower oxygen tensions (<1%), whereas 5% O_2_ would induce instead an initially low HIF-1 response followed by a delayed but higher HIF-2 activation [29]. To fully understand the transactivation capabilities of HIF family members in different oxygen tensions it would be necessary to use expression arrays or next gen sequencing techniques that would allow us to compare the induction of target genes under stress-inducing and physiological conditions.

The presence of an active HIF-1α suggested that it could be responsible for the induction of MAPK signalling at physiological oxygen tensions, which we previously described [4]. However, we found that ERK1/2 activation did not depend on the presence of HIF-1α in these conditions, in contrast to what has been described in hypoxic stresses [19]. Also, HIF-1α did not protect cells against DNA damaging agents, which we have shown is one of the effects of MAPK at these oxygen levels [4]. The mechanism by which MAPK signalling is induced in response to changes in oxygen levels remains to be elucidated. Of note, we found that inhibition of MAPK significantly impaired the activation of HIF-1α at 5% O_2_ in HCT116, placing it upstream of HIF-1α. This could be a specific feature of these cells, which have a hyperactive MAPK pathway due to overexpression of a mutant K-ras. Nevertheless, it is consistent with previous reports indicating that MAPK affects HIF-1α stabilization and activity in hypoxic stresses [9], [11] [18].

The impact on tissue homeostasis of the presence of HIF-1α at physiological oxygen tensions is likely to be very complex. We found that one of its main consequences is a notable increase in proliferation. Our experiments show that in the absence of HIF-1α, the elevated cell growth rates usually observed at physiological oxygen tensions [3], [4] were severely reduced. This supports our hypothesis that human cells *in vivo* are constitutively subjected to a series of signals that help them thrive in microenvironments with limitations in critical factors, such as oxygen [4]. The fact that most laboratory experiments are conducted at abnormally high oxygen levels (∼20%, traditionally called normoxia, despite the confusion that this may cause) prevents us from fully understanding these responses. We propose that it is important to differentiate the activity of the high levels of HIF-1 induced under stress situations (hypoxia and anoxia) and that of the low levels likely to be present in most tissues *in vivo*, which trigger distinct cellular responses (Figure 5E).

We also found that doxorubicin inhibited HIF-1α protein expression. It has been previously shown that doxorubicin can interfere with HIF-1 activity through an increase in the levels of p53 and its binding to p300 [22], and it has been suggested that p53 could decrease HIF-1α protein levels as well as its activity [23]. However, p53 was not responsible for the inhibition of HIF-1α in our experiments, as we also observed it in cells that lacked it. These results could help explain doxorubicin-mediated cardiac toxicity, a well-known side effect related to the oxidative stress induced by the drug [30], since heart tissue is highly sensitive to oxygen deprivation and blocking HIF-1α could impair the response of myocytes to stress. It is also important to consider a recent report that showed that doxorubicin increased HIF-1α expression in a tumour mouse model through a STAT1-nitric oxide dependent mechanism [31], which suggests that certain cancer cells may have a different response to doxorubicin *in vivo*.

Of note, DNA damaging agent actinomycin D was also capable of inhibiting HIF-1α, but the fact that others, such as ionizing radiation and oxidative stress, did not suggests that it is not a generic response to genotoxic stresses. Instead, HIF-1α inhibition may be a specific effect of certain drugs. Also, we showed that it is not limited to physiological oxygen tensions, since we could also observe it under severe hypoxic conditions. It would be interesting to test a wider range of DNA damaging agents in order to determine which of them have the ability to block HIF-1α expression and whether this plays any role in their known cytotoxic and antitumoural effects. It has been proposed that HIF-1α inhibition could be an effective strategy to block tumour progression and clinical trials are underway to test novel compounds that could do so [32]. Our results suggest that drugs that are currently being used in chemotherapy may already have that capability, besides their genotoxic properties. This information could help design improved antineoplastic strategies.

## Acknowledgments

We thank S. Cowley for useful discussions and critical reading of the manuscript.

## Supporting Information

Figure S1 **(A)** Western blot of HIF-2a levels in HCT116 cells incubated for 3 days at 20% O_2_ (20) or 5% O_2_ (5), also in the presence of 1.25 µM U0126 (5+U). (B) mRNA expression of GLUT-1 as measured by quantitative RT-PCR in HCT16 cells cultured at 20% or 5% O_2_ for 12 or 24 hours in the presence of 1 µM MEK inhibitor AZ6244. (C) mRNA expression of HIF-1α as measured by quantitative RT-PCR in HCT16 cells cultured at 20% or 5% O_2_ for 24 hours in the presence of 1.25 µM U0126, 0.4 µg/ml doxorubicin (Doxo) or nothing (C).
(TIF)

Figure S2 **Representative immunofluorescent images of EdU and DAPI-stained HCT116 HIF^+/+^ and HIF^−/−^.** Magnification: 20×.
(TIF)
