# Supplementary figures and images for: The Role of the HIF-1α Transcription Factor in Increased Cell Division at Physiological Oxygen Tensions
Source: PLoS One. 2014 May 16;9(5):e97938. doi: 10.1371/journal.pone.0097938 (PMC4024011; doi:10.1371/journal.pone.0097938)

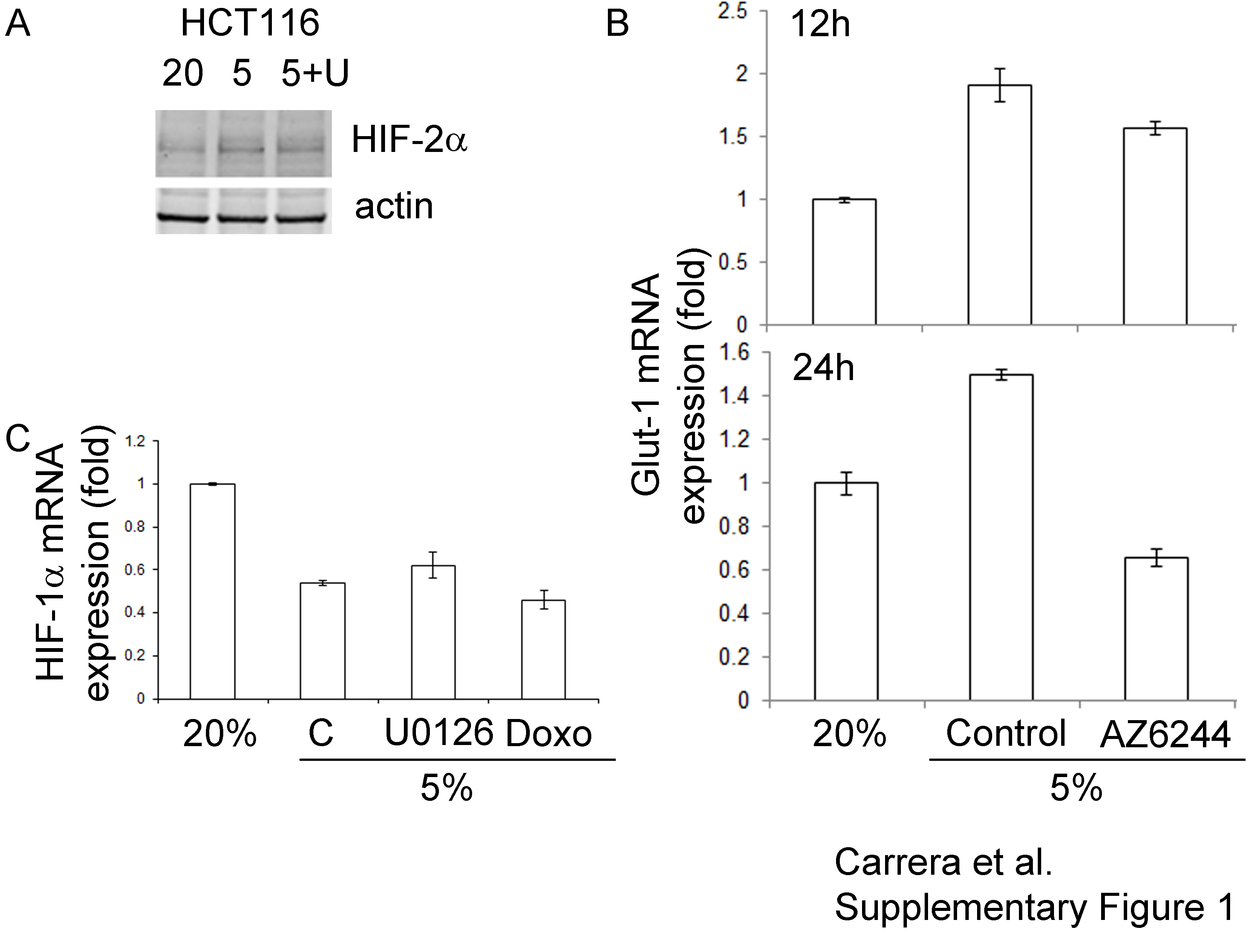

Supplement: Figure S1 — (A) Western blot of HIF-2a levels in HCT116 cells incubated for 3 days at 20% O2 (20) or 5% O2 (5), also in the presence of 1.25 µM U0126 (5+U). (B) mRNA expression of GLUT-1 as measured by quantitative RT-PCR in HCT16 cells cultured at 20% or 5% O2 for 12 or 24 hours in the presence of 1 µM MEK inhibitor AZ6244. (C) mRNA expression of HIF-1α as measured by quantitative RT-PCR in HCT16 cells cultured at 20% or 5% O2 for 24 hours in the presence of 1.25 µM U0126, 0.4 µg/ml doxorubicin (Doxo) or nothing (C). (TIF) [file pone.0097938.s001.tif]

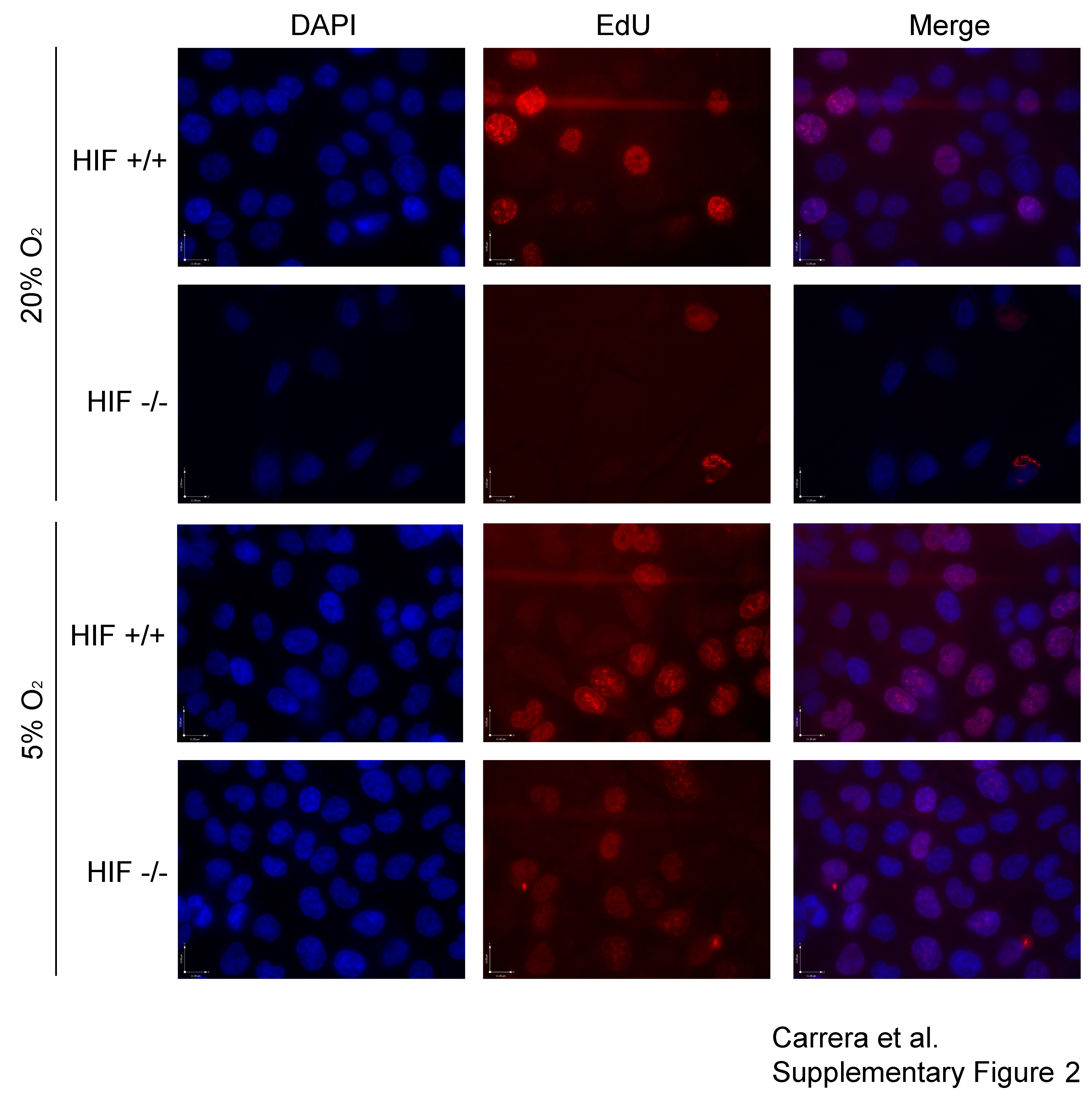

Supplement: Figure S2 — Representative immunofluorescent images of EdU and DAPI-stained HCT116 HIF+/+ and HIF−/−. Magnification: 20×. (TIF) [file pone.0097938.s002.tif]
